# An advanced three-dimensional phenotypic measurement approach for extracting *Ginkgo* root structural parameters based on terrestrial laser scanning

**DOI:** 10.3389/fpls.2024.1356078

**Published:** 2024-07-25

**Authors:** Yinyin Liang, Kai Zhou, Lin Cao

**Affiliations:** Co-Innovation Center for Sustainable Forestry in Southern China, Nanjing Forestry University, Nanjing, China

**Keywords:** root phenotyping, LiDAR, 3D reconstruction, *Ginkgo*, root structural parameters

## Abstract

The phenotyping of plant roots is essential for improving plant productivity and adaptation. However, traditional techniques for assembling root phenotyping information are limited and often labor-intensive, especially for woody plants. In this study, an advanced approach called accurate and detailed quantitative structure model-based (AdQSM-based) root phenotypic measurement (ARPM) was developed to automatically extract phenotypes from *Ginkgo* tree root systems. The approach involves three-dimensional (3D) reconstruction of the point cloud obtained from terrestrial laser scanning (TLS) to extract key phenotypic parameters, including root diameter (RD), length, surface area, and volume. To evaluate the proposed method, two approaches [minimum spanning tree (MST)-based and triangulated irregular network (TIN)-based] were used to reconstruct the *Ginkgo* root systems from point clouds, and the number of lateral roots along with RD were extracted and compared with traditional methods. The results indicated that the RD extracted directly from point clouds [coefficient of determination (*R* ^2^) = 0.99, root-mean-square error (RMSE) = 0.41 cm] outperformed the results of 3D models (MST-based: *R* ^2^ = 0.71, RMSE = 2.20 cm; TIN-based: *R* ^2^ = 0.54, RMSE = 2.80 cm). Additionally, the MST-based model (F1 = 0.81) outperformed the TIN-based model (F1 = 0.80) in detecting the number of first-order and second-order lateral roots. Each phenotyping trait fluctuated with a different cloud parameter (CP), and the CP value of 0.002 (*r* = 0.94, *p* < 0.01) was found to be advantageous for better extraction of structural phenotypes. This study has helped with the extraction and quantitative analysis of root phenotypes and enhanced our understanding of the relationship between architectural parameters and corresponding physiological functions of tree roots.

## 1 Introduction

Roots play a key role in supporting trees and the global carbon cycle, and they can also regulate ecosystem processes via plant–soil–microbe interactions by driving plants to obtain water and nutrients (Lynch, 1995; Rajendra et al., 2014; Villordon et al., 2014; Freschet et al., 2021b). Root system architecture (RSA) has become the “second green revolution” for global food security (Lynch, 2007). Structural and morphological characteristics, such as root diameter (RD), number, and lateral root isometry, are vital to understanding plant physiological functions (Gu et al., 2014; Wang, 2017; Seethepalli et al., 2021). Root phenotyping can track these structural and morphological characteristics and, thus, has great potential for bioenergy agroecosystems (York et al., 2022). Because of the challenge of directly gathering information on the roots underground, there are currently limited studies focusing on root phenotyping (Wilhelm et al., 2022).

Since the 1990s, innovative techniques and devices have been applied to root measurement, including non-destructive, manual, or automatic two-dimensional (2D) and three-dimensional (3D) digitizing techniques (Danjon and Reubens, 2008). Current research on root phenotyping primarily focuses on the cultivation of crops (e.g., rice, corn, and wheat), with higher yields, higher quality, and more excellent resistance to stress in the combination with genomic data (de Dorlodot et al., 2007; Kuijken et al., 2015; Topp et al., 2016; Tracy et al., 2020). The most extensively used method for root phenotyping is based on 2D images (Chen et al., 2018). However, 2D measurements are limited by the fact that pictures are typically taken from just one or two perspectives, where information can be lost as a result of roots overlapping (Shao et al., 2021). Traditional techniques for assembling root phenotyping information include minirhizotron techniques (Volkmar, 1993) and the agar gel culture method (Iyer-Pascuzzi et al., 2010). These methods are not only time-consuming and laborious, but also unable to describe the actual 3D structure of the root system (Delory et al., 2022). To further understand trait–function interactions, standardized and high-throughput approaches for acquiring root phenotypes are required (Wen et al., 2015; Delory et al., 2022). However, to the best of our knowledge, research on the 3D root structure of woody plants is still in its infancy (Li et al., 2015). In particular, there needs a systemic approach to accessing the architecture of tree roots (Zanetti et al., 2015). Although it is challenging to collect phenotypic information on the tree root system, evaluating the 3D root structure of woody perennials is crucial for understanding ecology (Liu, 1998), physiology and biochemistry (Steingrobe, 2001), morphology (Xi, 2019), biomechanics (Song et al., 2008), and bioenergy (York et al., 2022). More importantly, extracting tree root phenotyping traits is also critical in cultivating tree species with higher economic and ecological merits.

Structure from motion (SfM) has recently emerged as a digital tool for studying root structures (Koeser et al., 2016). This technique involves the acquisition of target point cloud data through photography, which is then used to perform 3D reconstruction (Lu et al., 2021). However, the performance of this method is influenced by the size of the object and the distance of measurement, and the process of dealing with background noise can be time-consuming (Okamoto et al., 2022). Computed tomography (CT) (Shao et al., 2021) and magnetic resonance imaging (MRI) (van Dusschoten et al., 2016) are currently popular techniques for the determination of 3D phenotypic information of roots, but less frequently to large woody root systems (Wu et al., 2021b). By digging up *Quercus petraea* and *Pinus pinaster*, Danjon et al. (1999) manually measured the diameter and topology of the root system and then reconstructed the 3D structure of the roots. However, manual measurements occupied an average of 2 to 3 h for each root. Danjon et al. (2005) adopted a 3D digitizer to measure the root structure of *P. pinaster*, reconstructing the 3D model of the roots and coloring the roots hierarchically, to link the structural properties of the roots with the stability of against wind. Yang (2021) used SketchUp software to simulate the 3D visualization of the root system of slope protection plants. The root configuration parameters, topological indexes, and fractal dimensions were extracted, which provided an important basis for the planting method and species selection of slope protection plants. However, owing to the time-consuming and laborious recording of coordinates, diameter, angle, and other parameters, the 3D structure of the root system cannot be directly obtained. Zhang et al. (2020) applied time-consuming 3D printing to simulate the 3D structure of roots with a physical model. This model utilized four fixed-sized RDs to represent the entire root system, which hardly capture the real RSA and morphology of roots. Spanos et al. (2008) obtained root structure information by uprooting *Abies cephalonica* Loudon with a 3D digitizer, which was limited to the lab analysis. Zhang et al. (2021) utilized ground-penetrating radar (GPR) to detect the roots of *Pinus sylvestris* var. *mongolica*, by connecting the root system’s coordinate to determine its spatial distribution. Because of the influence of soil water content and resolution, as an emerging nondestructive detection technique, GPR cannot detect fine roots (RD less than 2 mm) and cannot directly obtain the 3D structure of roots. Quantitatively obtaining multidimensional information on plant roots, for constructing 3D models with a high efficiency, has become a challenging problem in root visualization research (Wu et al., 2021a).

Light detection and ranging (LiDAR) is a fast, non-destructive, and accurate remote sensing sensor for monitoring plant information (Cao et al., 2014; Lin, 2015; Zhou and Cao, 2021). Terrestrial laser scanning (TLS), a near-ground active remote sensing technique (Raumonen et al., 2013), can efficiently and accurately gather information about 3D point clouds of trees (Wang et al., 2021). It can also quantitatively extract the parameters and skeleton of trees for creating 3D models (Liu, 2016). Previous studies focused primarily on the aboveground components (e.g., branches, leaves, and trunks) (Liu et al., 2016; Disney, 2019), but only a few on roots underground with different typical root phenotyping acquisition methods and sensors (**Table 1**). Smith et al. (2014) and Todo et al. (2021) demonstrated that TLS point clouds are capable of accurately representing tree root architecture, thereby providing a robust technical foundation for 3D models to characterize root variables. *Ginkgo* (*Ginkgo biloba* L.) is a deep-rooted tree species (Fu and Zhang, 2019) and an essential economic tree species in China, with various valuable characteristics, namely, medicinal, edible, and ecological, and it can also be used in landscaping (Shen et al., 2020). Research on *Ginkgo* now focuses on the aboveground part, and it is uncommon to find studies on its root systems (Men, 1986). Studies on the morphology and structure of the *Ginkgo* root system, as well as the establishment of 3D models, can contribute to understanding its physiological activities and mechanisms. Additionally, genetic data can also be combined with root phenotyping to create more tolerant and productive plants. A sound and organized system of plant research is made possible by quantitative descriptions of the root structural parameters.

**Table 1:** Comparison of root phenotyping acquisition methods with different sensors.

Data type	Sensor	Species	Advantages of techniques	Disadvantages of techniques	References
**2-D**	RGB camera	Crops, herbs	High throughput, low cost	Large amount of data, incomplete root system image information	(Yin et al., 2009); (Wilhelm et al., 2022)
	Electrical resistance tomography (ERT)	Trees	Non-destructive	Multiple sources of error, highly influenced by soil moisture	(Amato et al., 2008); (Zhao et al., 2019)
**3-D**	3-D digitizer	Trees, crops	High precision, semi-automatic	Time-consuming, complicated operation	(Danjon et al., 1999); (Spanos et al., 2008)
	X-ray computed tomography (CT)	Crops	Non-destructive, high precision	Costly, unable to detect coarse roots, indoor operation	(Perret et al., 2007); (Shao et al., 2021)
	Laser scanning (LiDAR)	Trees	Wide range of detection, high precision	Costly, uneven point cloud density	(Smith et al., 2014); (Todo et al., 2021)
	Magnetic resonance imaging (MRI)	Crops	Non-destructive, high precision	Costly, unable to detect coarse roots, indoor operation	(van Dusschoten et al., 2016)
	Ground-penetrating radar (GPR)	Trees	Non-destructive, *in situ* detection	Low resolution, unable to detect fine roots, highly influenced by soil and water	(Zhang et al., 2020); (Alani and Lantini, 2020)

However, to date, limited comparable investigations utilizing LiDAR have been carried out on the root systems of woody plants. It would be of high potential value for parameterizing 3D models of tree root systems to quantify the relationship between RD and other root phenotyping traits. Therefore, in this study, we utilized TLS to scan the *Ginkgo* root systems for obtaining its 3D structure, while establishing 3D models of the root system and automatically extracting several phenotyping traits. Specifically, the objectives of this study were (1) to develop an advanced approach of accurate and detailed quantitative structure model-based (AdQSM-based) root phenotypic measurement (ARPM) for extracting 3D phenotypic parameters of tree roots, (2) to evaluate the ability of the developed approach for extracting structural parameters of *Ginkgo* roots, and (3) to analyze the variations of the *Ginkgo* root structural parameters automatically extracted based on the developed approach by considering different parameters.

## 2 Materials and methods

**Figure 1:**
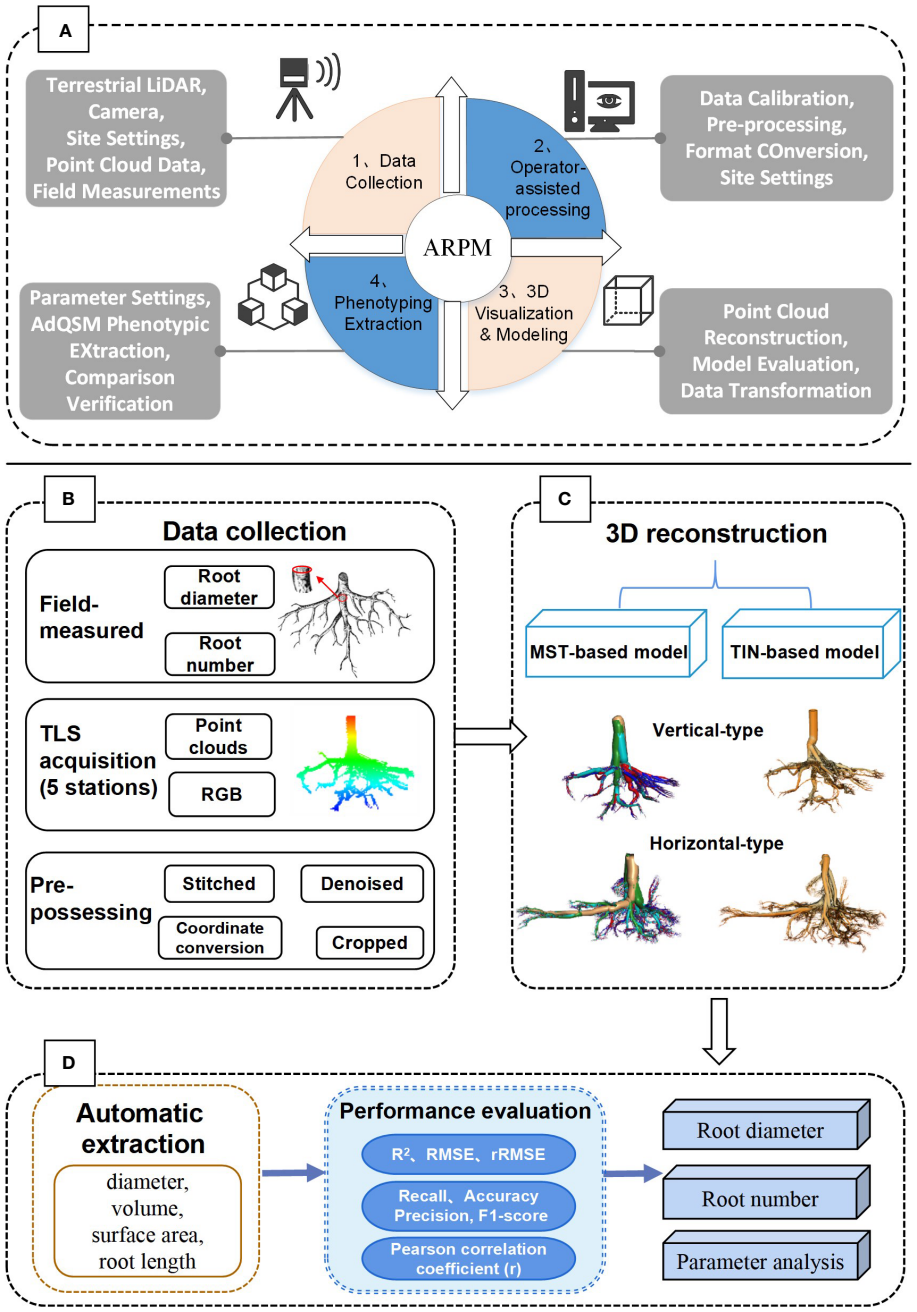
The overall process framework of the study. **(A)** The proposed ARPM approach for phenotypic measurement and extraction of tree roots, which includes four steps: data collection, operator-assisted processing, 3D visualization modeling, and phenotyping extraction. **(B)** Data collection and processing steps. **(C)** Methods of 3D reconstruction modeling. **(D)** Parameter extraction and performance evaluation.

**Figure 1** shows the overall framework for measuring Ginkgo root phenotypes, which includes the operation of the developed ARPM approach (A), as well as the specific workflow of the study (B). The ARPM is divided into four modules: data acquisition, operator-assisted processing, 3D visualization and modeling, and phenotyping extraction. Specifically, LiDAR data were collected by setting up site scans, followed by preprocessing such as stitching, denoising, cropping, and coordinate conversion. Secondly, two approaches were further used to reconstruct the root: minimum spanning tree (MST)-based and triangulated irregular network (TIN)-based models. Accordingly, we employed various metrics to assess the models’ performance. The coefficient of determination (*R* ^2^), root-mean-square error (RMSE), and mean absolute error (MAE), for example, are used to assess the RD; Recall, Precision, F1-score, and Accuracy are used to assess the number of roots. Thirdly, we used the AdQSM algorithm to automatically extract essential root traits and the Pearson correlation coefficient to assess the relationship between aspiration rate and RD.

### 2.1 Data acquisition

The information was gathered on 27 December 2021 at the Xiashu Experimental Forestry Site of Nanjing Forestry University, Jiangsu Province (119°22′E, 32°12′N). The climate of the study area is northern subtropical monsoon climate, with an average annual temperature of 15.5°C, an average annual precipitation of 1,099.1 mm, and a landscape of hilly areas. The plot size is 20 m × 20 m with a tree density of 1,475 trees/ha. The *Ginkgo* trees in the sample plot are planted artificially, and the average age of the trees is 22 years old. Before selecting the sample trees, we considered the tree height, diameter at breast height (DBH), and uprightness of the trees in the plot. We selected six trees with good growth and upright trunks, labeled a, b, c, d, e, and f, based on a combination of three sizes of DBH (>12 cm, 9–12 cm, and 6–9 cm). The roots of the trees were cleaned and manicured before being raised entirely by an excavator and marked with red paint precisely for pointing the south direction of the trunk. The 22-year-old *Ginkgo* tree has a deep root system (root depth approximately 2 m), which causes its roots to be buried deep underground for a very long period. Given this fact, the surface of the roots is tangled up with soil and fine roots, making it challenging to determine the topological structure of the roots. The fine roots were cut back to highlight the RSA. Coarse roots with a base diameter larger than 0.5 cm were measured.

The TLS, which combines LiDAR and a digital camera, is one of the more crucial pieces of hardware in the ARPM. The fixed *Ginkgo* trees’ roots were scanned using the RIEGL VZ-400i Terrestrial Laser Scanning (RIEGL Laser Measurement Systems, Inc., Horn, Austria) and integrated with a Nikon D810 camera (resolution: 7,380 × 4,928 pixels) to produce true color images and high-density 3D point clouds. The device has a measurement accuracy of ≤5 mm, a range of 800 m, a field of view of 100° × 360° (vertical × horizontal), and a maximum laser pulse repetition rate of up to 1.2 MHz. The angular resolution was set to 0.0007°C and 0.0005°C for the vertical and horizontal angles, respectively. Five different scanning positions were evenly distributed around the target in the center, with an interval angle of approximately 70°C.

### 2.2 Data processing

#### 2.2.1 Preprocessing

In this study, the ARPM approach provided the first attempt to use TLS as a sensor to obtain 3D point clouds of the roots. To achieve automatic site data stitching, the raw data from TLS scanning are fed into the accompanying RiSCAN PRO software (http://www.riegl.com/products/software-packages/riscan-pro/), with a registration error of less than 2 mm (Henning and Radtke, 2008). ICP (iterative closest point) is the foundation of the point clouds stitching algorithm. If the automatic stitching effect is inadequate, fine-tune manually to get each site point cloud as tightly fitted as possible. The TLS is equipped with a digital camera with a fixed focal length to acquire texture information from the target object’s surface. The stitched point clouds were imported into LiDAR360 software (Beijing Green Valley Technology. Co., Ltd., China, https://www.lidar360.com), and the root point clouds were cropped out separately and then denoised. Usually, modeling is bottom-up; in terms of morphology, it is from the apical side to the basal side. Aboveground branches and underground roots are similar in morphology that they are classified as trunk or taproot, first-order lateral, second-order lateral, etc. Furthermore, given underground roots do not have leaves, the noise source and shielding area are reduced as compared to aboveground modeling. Hence, we proposed and tested a hypothesis for conducting bottom-up branch separation to root reconstruction and extraction. Since the root system is oriented from the root base to the root tip, the typical upright root systems need to be inverted for further modeling.

#### 2.2.2 3D reconstruction

**Figure 2:**
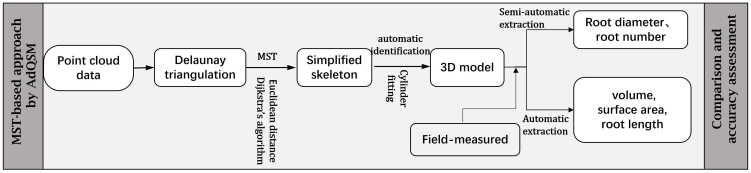
Flowchart of the MST-based approach by AdQSM for phenotypes measurement of *Ginkgo* roots. The point cloud data underwent initial Delaunay triangulation, followed by simplification using specific algorithms aimed at streamlining the skeleton. Subsequently, the simplified skeleton was molded to resemble cylinders to obtain a 3D model. Finally, the model was assessed through the extraction of root system parameters.

**Figure 3:**
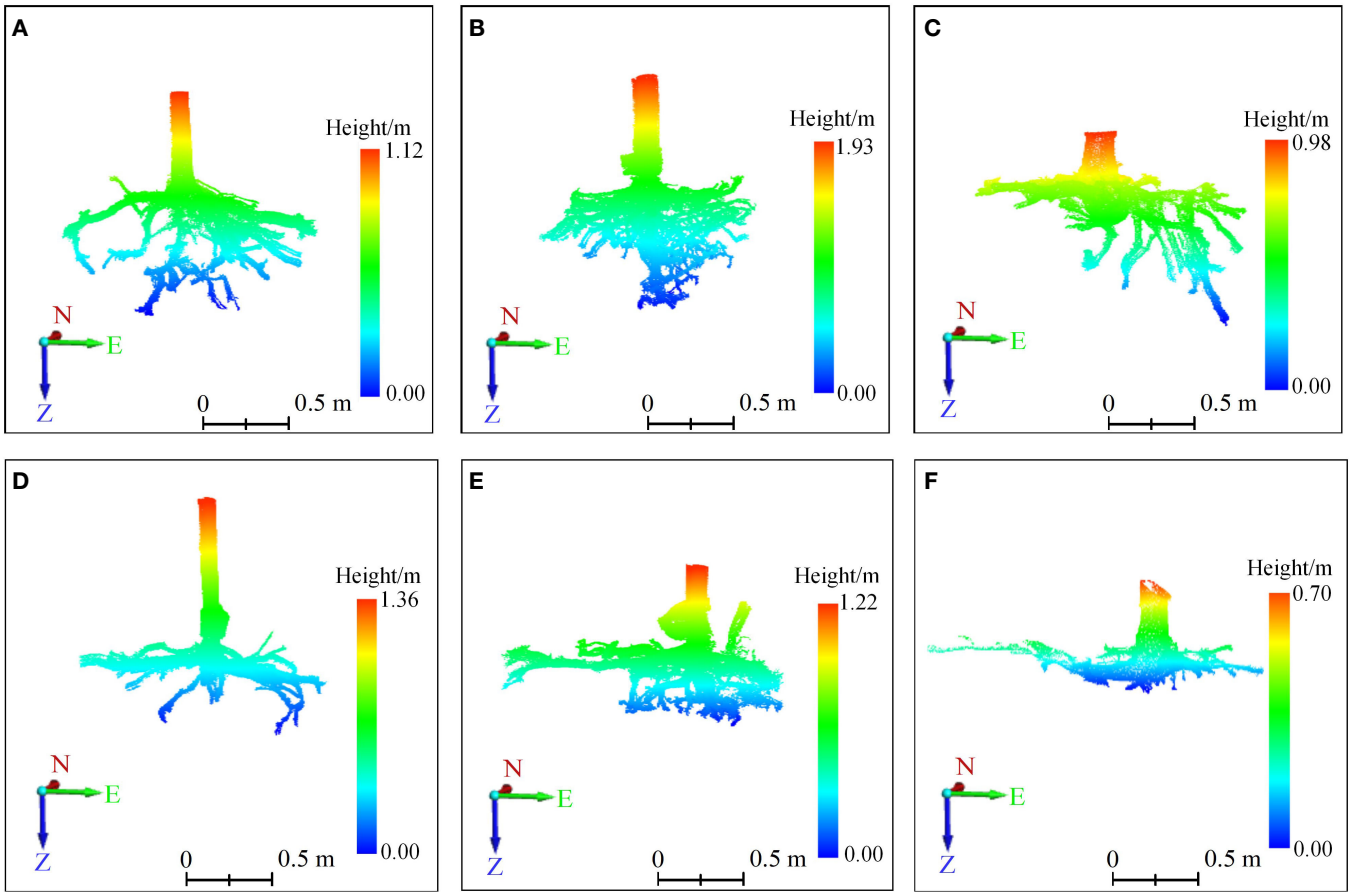
The 3D point clouds’ front view of root systems. **(A–F)** correspond to six sample trees, with those labeled **(A–C)** indicating trees exhibiting vertical root growth, and those labeled **(D–F)** indicating trees with horizontal root growth. On the coordinate axis, N represents north, E stands for east, and Z indicates the direction of root growth. The root systems were scanned using TLS to generate point clouds, which were displayed after preprocessing steps like stitching, cropping, and denoising.

**Figure 4:**
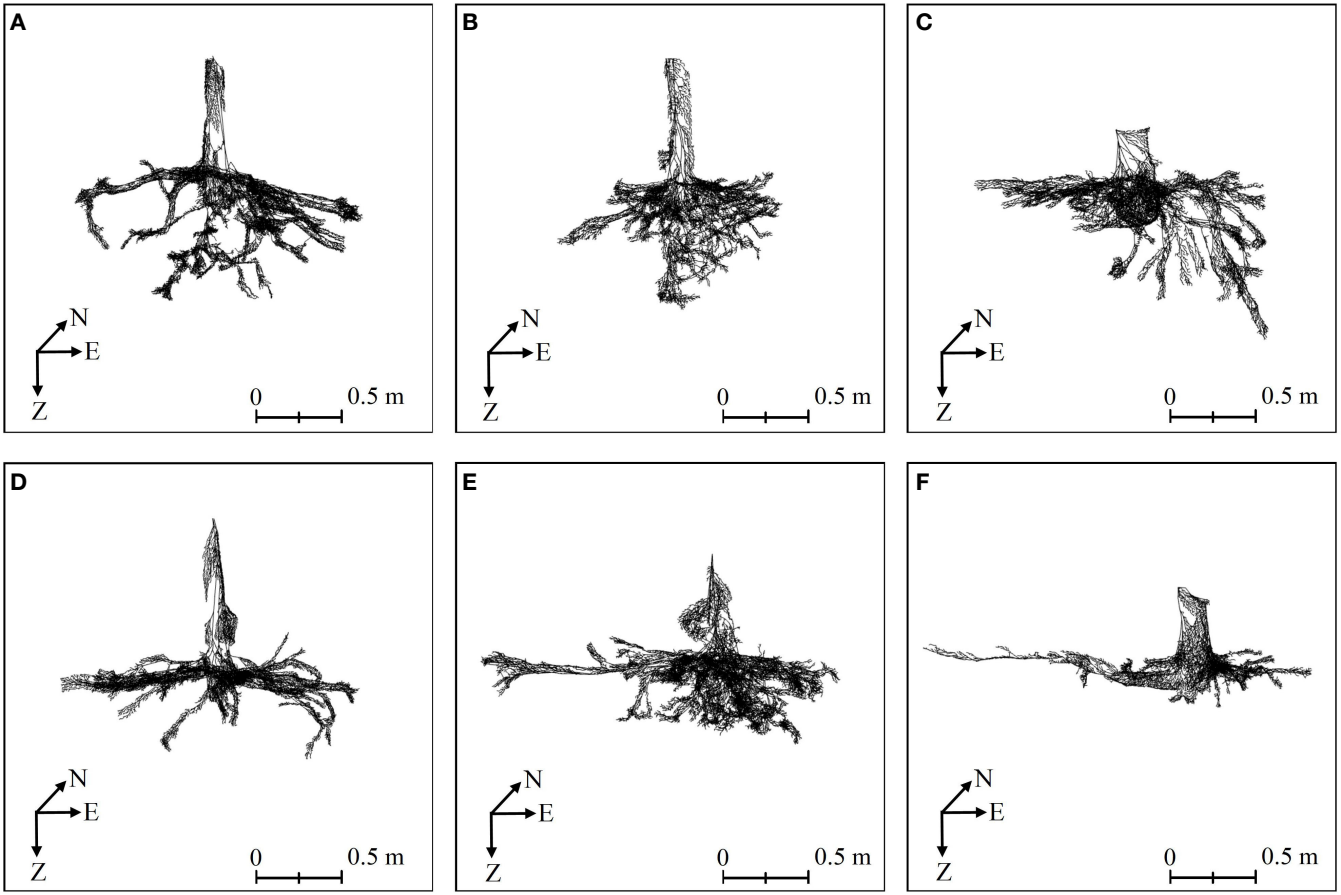
The root skeletons of six Ginkgo trees obtained by MST-based algorithm in AdQSM. **(A–F)** correspond to six sample trees. On the coordinate axis, N represents north, E stands for east, and Z indicates the direction of root growth.

**Figure 5:**
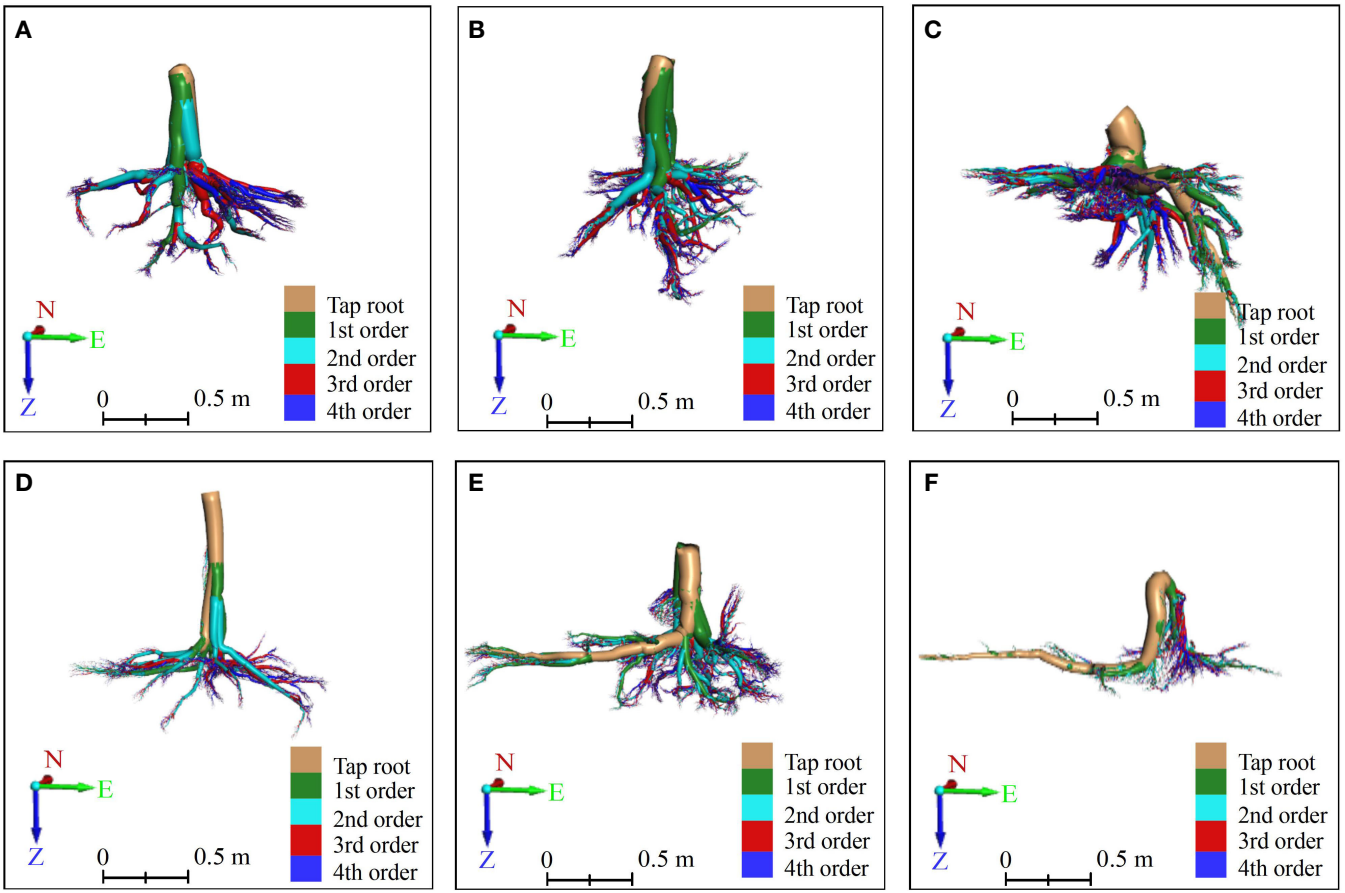
The front view of vertical-type **(A–C)** and horizontal-type **(D–F)** Ginkgo root systems, which were constructed by an AdQSM-based approach. The method reconstructed the roots in a bottom-up manner according to its growth rules, and calculated each branch node and order to grade the roots. Different orders of roots are colored by various colors. The tap roots, the first-order (1st) lateral roots, the second-order (2nd) lateral roots, the third-order (3rd) lateral roots, and the fourth-order (4th) lateral roots are colored brown, green, cyan, red, and blue, respectively. On the coordinate axis, N represents north, E stands for east, and Z indicates the direction of root growth.

Three-dimensional quantitative structural modeling (3D QSM) can contribute to the knowledge of spatial distribution characteristics, traits, and growth of the root system (Smith et al., 2014). 3D reconstruction is a critical step in the developed ARPM. The developed ARPM takes 10 min to capture point clouds of one entire root system, and the visualization is done on the computer side by reading the data. The pipeline of the 3D reconstruction approaches is shown in **Figure 1**. For the plant reconstruction, there are mainly two approaches based on segmentation and skeleton (Raumonen et al., 2013). In this study, we primarily employ two different approaches to extract the skeleton and then generate the root model automatically. The first approach is MST-based, and the second is TIN-based. AdQSM is a method developed by improving on AdTree (Du et al., 2019) and TreeQSM (Raumonen et al., 2013). The workflow of AdQSM is shown in **Figure 2**. **Figure 3** shows the front view of the processed TLS 3D point clouds of the Ginkgo root system. The key algorithm is to construct the MST using Dijkstra’s shortest path algorithm (Dijkstra, 1959) to obtain the skeleton of a tree (**Figure 4**). The branches are then reconstructed based on K-means clustering and nonlinear least squares optimized cylinder fitting with the aim of obtaining a more refined geometric structure model (Fan, 2021). Tree roots can be divided into tap roots, first-order lateral roots, second-order lateral roots, etc (Ingram and Malamy, 2010; Wang, 2017). To grade the roots and assign them a specific color, the process is recreated from the bottom up by computing each branch node and its order (**Figure 5**). The algorithm is robust to issues like missing or insufficient regional point clouds.

**Figure 6:**
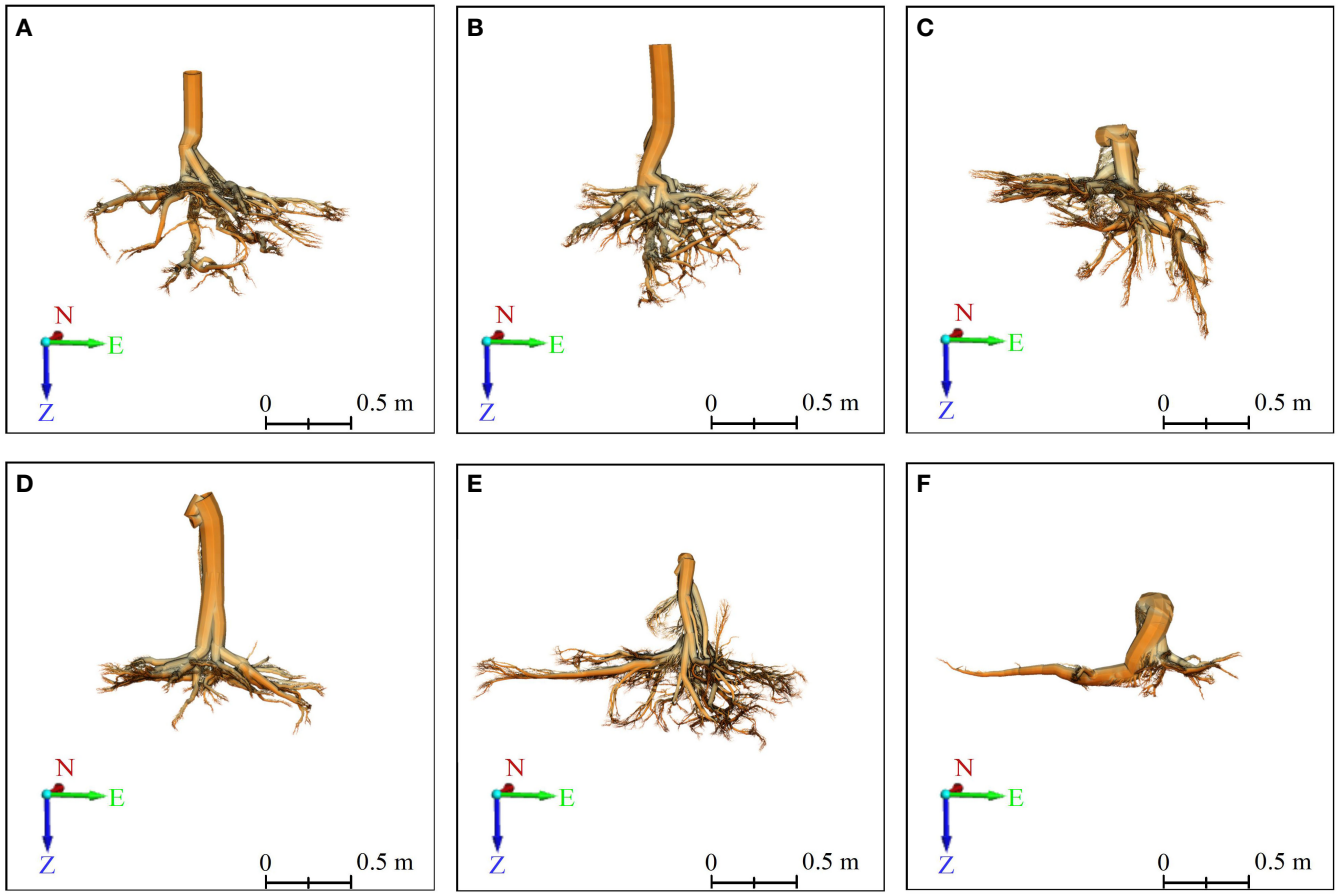
3D front view of six Ginkgo root systems, modeled by the TIN-based algorithm in PCAV, and CloudCompare presents the final model. **(A–F)** correspond to six sample trees, with those labeled **(A–C)** indicating trees exhibiting vertical root growth, and those labeled **(D–F)** indicating trees with horizontal root growth. On the coordinate axis, N represents north, E stands for east, and Z indicates the direction of root growth.

The Point Cloud Automata Viewer (PCAV) (Tianhong Jiye Technology Development Co., Ltd., China, http://www.thjymap.com/pca) filters point clouds based on the Multi-Primitive TIN Progressive Densification (MPTPD) algorithm of object primitives to automate modeling by generating a triangular mesh tree skeleton (Lin and Zhang, 2014; Lin et al., 2016). It is a commercial software, and its interface is developed based on the opensource project CloudCompare (https://www.cloudcompare.org) (Rajendra et al., 2014) the root model reconstructed by PCAV is shown in **Figure 6**. The TIN representation uses the discrete data obtained from all sampling points and connects these discrete points (vertices of triangles) into continuous triangular surfaces according to the principle of optimized combination. When constructing a TIN from the point cloud, the normal vector and centroid of each triangle in the TIN are calculated as follows (Equations 1, 2) (Wu et al., 2021c). Assume the vertices of triangle Q_i_ are G_1_ (X_1_, Y_1_, Z_1_), G_2_ (X_2_, Y_2_, Z_2_), and G_3_ (X_3_, Y_3_, Z_3_), in that order. The normal vector is N = (A, B, C), which can be expressed as the result of the cross-product between G_1_G_2_ and G_1_G_3_. The center of mass of triangle Q_i_ is C_k_. Calculate the vectors between G_1_ and G_2_, and between G_1_ and G_3_, as G_1_G_2_ = (X_2_ − X_1_, Y_2_ − Y_1_, Z_2_ − Z_1_) and G_1_G_3_ = (X_3_ − X_1_, Y_3_ − Y_1_, Z_3_ − Z_1_), respectively.


$$A\;=\;\left(Y_{2}\;-\;Y_{1}\right)\left(Z_{3}\;-\;Z_{1}\right)\;-\;\left(Z_{2}\;-\;Z_{1}\right)\left(Y{\;}_{3}-\;Y_{1}\right)$$

(1) $$B\;=\;\left(Z_{2}\;-\;Z_{1}\right)\left(X_{3}\;-\;X_{1}\right)\;-\;\left(X_{2}\;-\;X_{1}\right)\left(Z_{3}-\;Z_{1}\right)$$

$$C\;=\;\left(X_{2}\;-\;X_{1}\right)\left(Y_{3}\;-\;Y_{1}\right)\;-\;\left(Y_{2}\;-\;Y_{1}\right)\left(X_{3}-\;X_{1}\right)$$

The centroid C_k_ is calculated as follows:


(2) $$C_{k}\;=\left(\frac{X_{1}+X_{2}+\;X_{3}}{3},\frac{Y_{1}+Y_{2}+\;Y_{3}}{3},\;\frac{Z_{1}+Z_{2}+\;Z_{3}}{3}\right)$$

#### 2.2.3 Automatic extraction of phenotyping information

A quantitative understanding of the phenotyping traits of roots facilitates the understanding of the environmental–functional mechanisms of root action. In this study, multiple phenotyping traits (diameter, surface area, volume, and length) were extracted automatically using AdQSM v1.7 (open access: https://github.com/GuangpengFan/AdQSM) (Fan et al., 2020). The algorithms are modeled in a bottom-up manner to calculate the order and the number of bifurcation points of each grade of branches, which correspond to the grading number and the number of different grades of roots, respectively (Dong et al., 2021) (Ajmera et al., 2022). The branching order and basal diameter of the branches correspond to the grading order of the root system and the RD (Fan, 2021).

### 2.3 Model evaluation methods

#### 2.3.1 Root diameter accuracy evaluation method based on point clouds and models

To evaluate the accuracy of the model, the basal diameters of approximately 2–4 RDs were randomly selected for each sample tree (19 RDs in total) and compared with the diameters extracted from the point cloud and the model, respectively. RDs were measured with vernier calipers serving as the true values, while those extracted from point clouds and models are considered as extracted values. The point clouds of roots are first segmented in LiDAR360. To make the upper end of the root morphology vertical, the projection and coordinate transformation were applied to the segmented individual roots. The least squares circle fitting algorithm was used to measure its basal diameter, with the average of several measurements adopted to determine the diameter. Likewise, CloudCompare was used to crop the point clouds of the root bases of the reconstructed models, and then importing it into LiDAR360 to obtain the model extracted values of RD. The *R* ^2^, RMSE, relative root-mean-square error (rRMSE), and MAE were calculated to estimate the level of consistency between the point clouds, 3D models’ measurement data, and the raw data collected in the field (Equations 3–6). The metrics were calculated as follows:


(3) $$R^{2}=1-\frac{{\sum_{i=1}^{n}\left(x_{i}-{\hat{x}}_{i}\right)}^{2}}{{\sum_{i=1}^{n}\left(x_{i}-\bar{x}\right)}^{2}}$$

(4) $$RMSE=\sqrt{\frac{1}{n}{\sum_{i=1}^{n}\left(x_{i}-{\hat{x}}_{i}\right)}^{2}}$$

(5) $$rRMSE=\frac{RMSE}{\bar{x}}\times 100\%$$

(6) $$MAE=\frac{1}{n}\sum_{i=1}^{n}\left|{\hat{x}}_{i}-x_{i}\right|$$

where $x_{i}$ is the measured root diameter; $\bar{x}\;$ is the mean of the measured root diameter; ${\hat{x}}_{i}$ is the estimated value of the root diameter model; *n* is the number of samples.

#### 2.3.2 Accuracy evaluation methods for models to identify different levels of roots

The main traits to describe the root architecture are the number, diameter, and grade of the taproots and lateral roots (Xiao et al., 2014). From the 3D laser point clouds of *Ginkgo*, it can be found that its root type belongs to the horizontal or vertical root system (Zanetti et al., 2015; Xi, 2019), and the taproots are thick or not prominent. In this study, the number of first-order lateral roots (including the taproot) and second-order lateral roots was counted separately. To quantitatively distinguish taproots, first-order lateral roots, and second-order lateral roots, a computerized 3D visualization was utilized, combined with the visual interpretation of root point clouds or models. The accuracy of the models was evaluated by comparing it with the measured number of lateral roots measured by TLS. The performance of the model is evaluated using Recall, Precision, F1-score (F1), and Accuracy, all of which vary from 0 to 1 (Equations 7–10). The formulas are shown below, respectively.


(7) $$\text{Recall}=\frac{TP}{TP+FN}$$

(8) $$\text{Precision}=\frac{TP}{TP+FP}$$

(9) $$\text{F}1=2\times \frac{Recall\times Precision}{Recall+Precision}$$

(10) $$\text{Accuracy}=\frac{TP+TN}{TP+TN+FP+FN}$$

where TP, TN, FN, and FP refer to true positives, true negatives, false negatives, and false positives, respectively. F1-score is basically a harmonic mean of precision and recall.

#### 2.3.3 Evaluation for the automatically extracted parameters

The correlation between the automatically extracted parameters and the true values is calculated by the Pearson correlation coefficient (*r*) (Equation 11). The calculation formula of *r* is:


(11) $$r=\frac{\sum_{i=1}^{n}\left(x_{i}-\bar{x}\right)\left(y_{i}-\bar{y}\right)}{\sqrt{\sum_{i=1}^{n}{\left(x_{i}-\bar{x}\right)}^{2}\sum_{i=1}^{n}{\left(y_{i}-\bar{y}\right)}^{2}}}$$

where $x_{i}$ is the extracted parameter for the *i*th samples; $\bar{x}\;$ is the mean of $x_{i}$ ; $y_{i}$ is the true value (typically measured manually) for the *i*th samples; $\bar{y}\;$ is the mean of $y_{i}$ ; *n* is the number of samples. The coefficient *r* ranges from −1 to 1. If the absolute value of *r* is close to 1, the linear correlation between the extracted values and the real value is stronger; if it is 0, there is no correlation between them.

## 3 Results

### 3.1 Accuracy assessment of the root diameter

**Figure 7:**
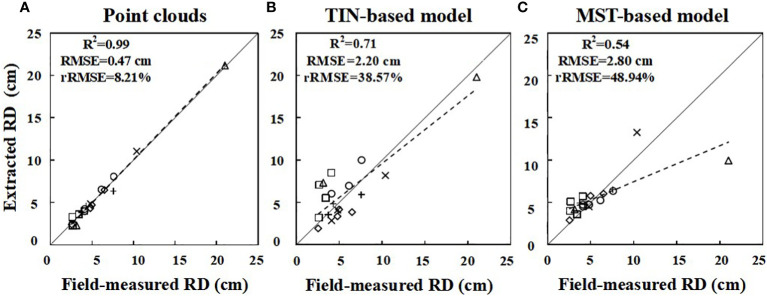
Linear fit of the root diameter (RD) extracted from point clouds **(A)** and the MST-based **(B)** and TIN-based **(C)** model to the measured values, respectively. The six symbols “□⋄△+○×” represent roots from A, B, C, D, E, and F, respectively. The solid line represents the 1:1 line, and the dashed line indicates the regression equation.

In this study, two root models were selected to extract 19 RDs from six *Ginkgo* trees. A linear regression was fitted between the measured diameter (manually measured with a vernier caliper) and the values extracted from the point cloud and models, and a scatter plot was drawn (**Figures 7A–C**). For extracted diameters, the root point clouds acquired by TLS (*R* ^2^ = 0.99, MAE = 0.35 cm, RMSE = 0.47 cm, rRMSE = 8.21%) are highly consistent with the manually measured values, which are more accurate than the rebuilt root models. For the reconstructed MST-based (*R* ^2^ = 0.71, MAE = 1.79 cm, RMSE = 2.20 cm, rRMSE = 38.57%) and TIN-based (*R* ^2^ = 0.54, MAE = 5.62 cm, RMSE = 2.80 cm, rRMSE = 48.94%) 3D models, the former fits better and has higher accuracy when it is compared with manual measurement values. The RMSE of the two models ranged from 2 to 3 cm. There is a point with a large deviation (**Figure 7C**). Given the fact that the tree labeled c has more fine roots, this large deviation is mostly caused by the underestimation of this high-diameter root, as well as some root scans are not of good enough quality for the point cloud, resulting in a biased reconstructed model. This value reflects the variation in RD extracted by different models, with higher values in the TIN-based model tending to become saturated.

### 3.2 Accuracy evaluation of the model to identify different orders of roots

**Figure 8:**
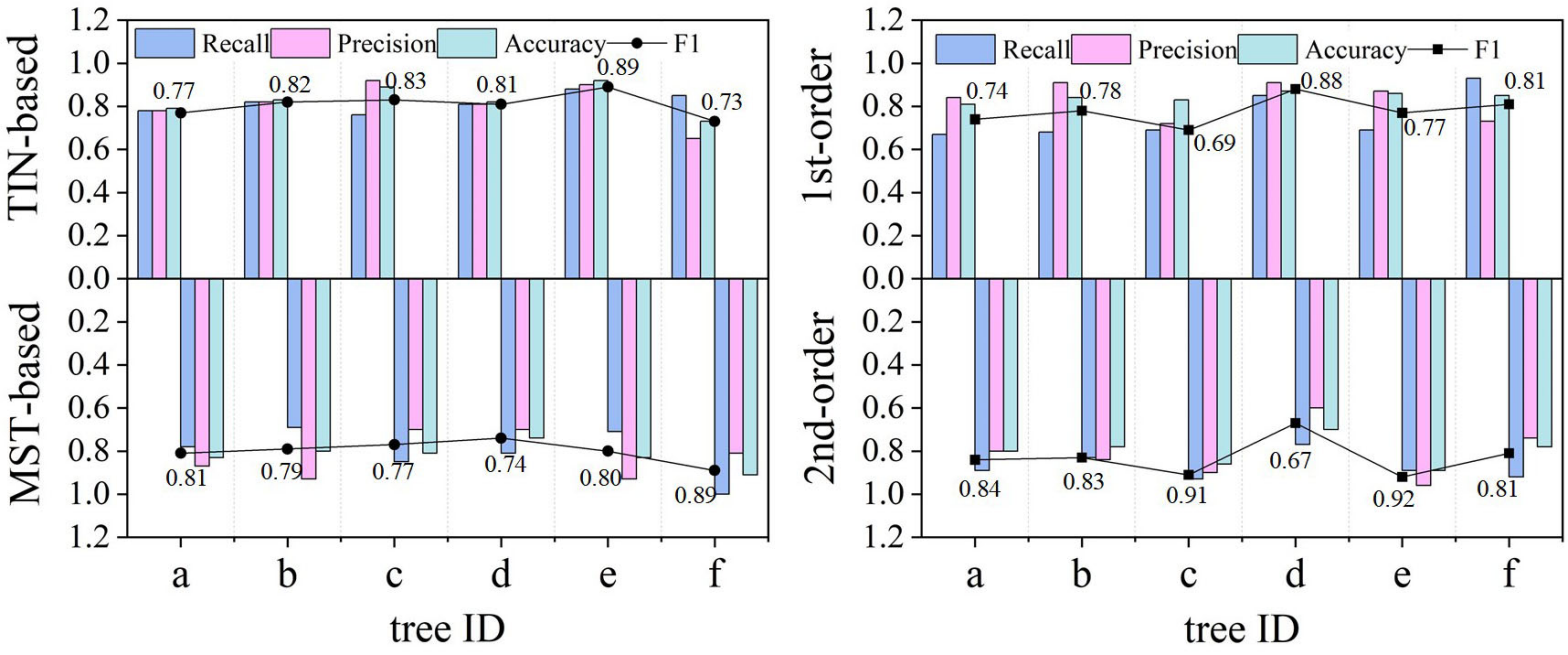
Comparison of the detection effect of the number of lateral roots from the two models regarding different evaluation metrics. The MST-based model (F1 = 0.81) surpassed the MST-based model (F1 = 0.80). The better performance of the second (2nd) order (F1 = 0.83) than the first (1st) order (F1 = 0.78) lateral roots is explained by the larger number of the former and their wider distribution, which makes them easier to be identified. Conversely, the 1st-order lateral roots are often misidentified (false negative) due to noise or occlusion.

**Table 2** demonstrates the accuracy of the two models for identifying different classes of root systems. In terms of the models’ detection of the number of roots, the MST-based model (F1 = 0.81, Accuracy = 0.83) possesses slightly higher overall accuracy than the TIN-based model (F1 = 0.80, Accuracy = 0.82), and both Recall and Precision are roughly comparable, with mean values of approximately 0.8. In particular, the second-order lateral roots (F1 = 0.83) were somewhat better than the first-order lateral roots (F1 = 0.78), in terms of the root number for model detection. Although the accuracy of the first-order lateral roots is higher than that of second-order lateral roots, this is due to the fact that the number of second-order roots is typically more than that of the first-order roots, and an imbalanced distribution will impair the accuracy outcomes. Additionally, **Figure 8** visualizes the comparison between Recall, Precision, F1, and Accuracy for the MST-based and TIN-based models, and the first- and second-order lateral roots.

**Table 2:** Overall accuracy assessment of the two models for identifying different orders of root systems.

Evaluation index	The first-order root		The second-order root		Overall evaluation	
	MST-based	TIN-based	MST-based	TIN-based	MST-based	TIN-based
**Recall**	0.76	0.75	0.87	0.87	0.82	0.81
**Precision**	0.83	0.83	0.80	0.81	0.81	0.82
**F1-score**	0.79	0.77	0.83	0.83	0.81	0.80
**Accuracy**	0.86	0.84	0.81	0.80	0.83	0.82

### 3.3 Results of automatic phenotype extraction

**Figure 9:**
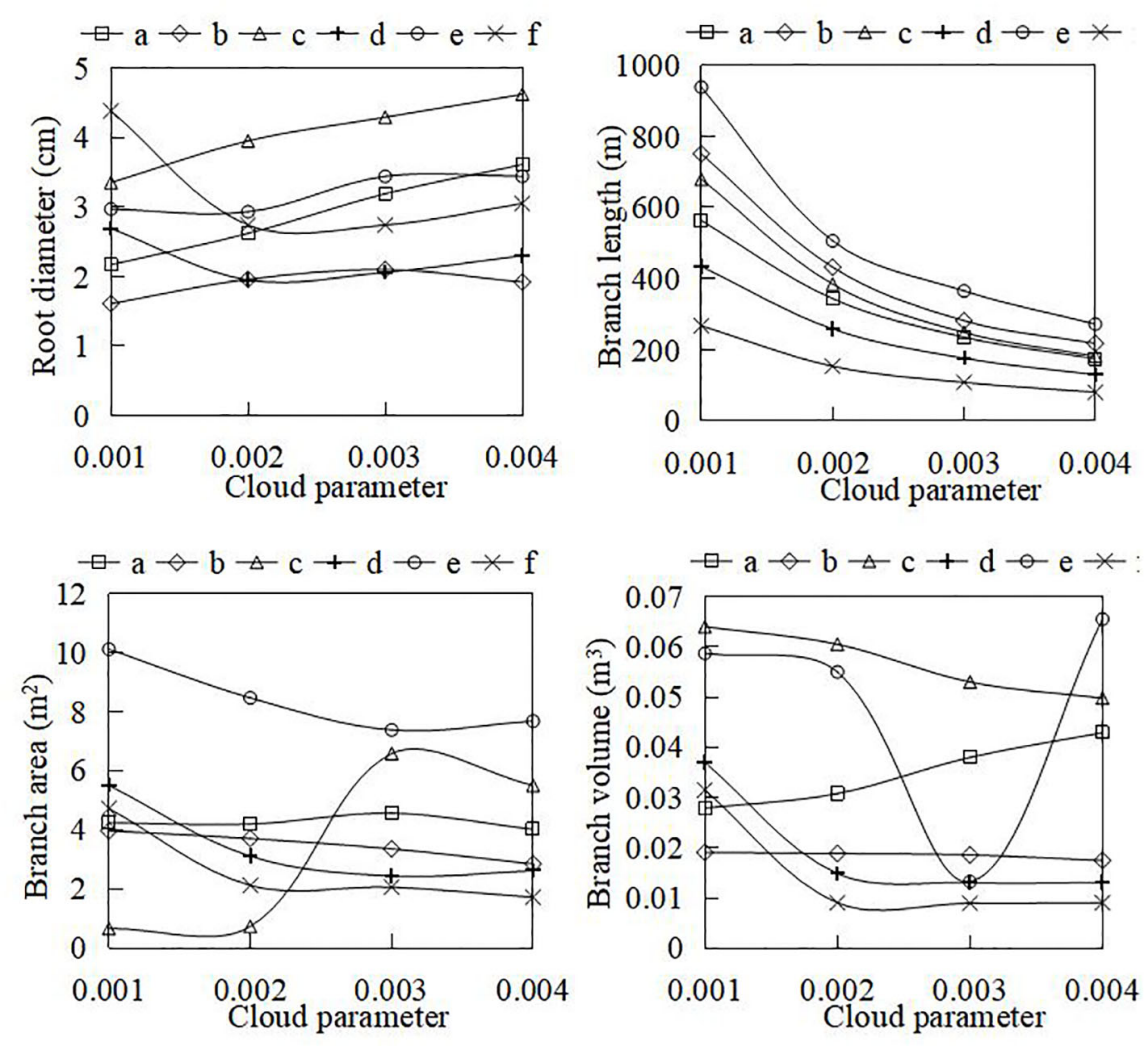
The variation of automatically extracted parameters (e.g., root diameter, branch volume, branch surface area, and branch length) at different cloud parameter (CP) coefficients. The CP represents the dilution rate during point cloud processing.

**Figure 10:**
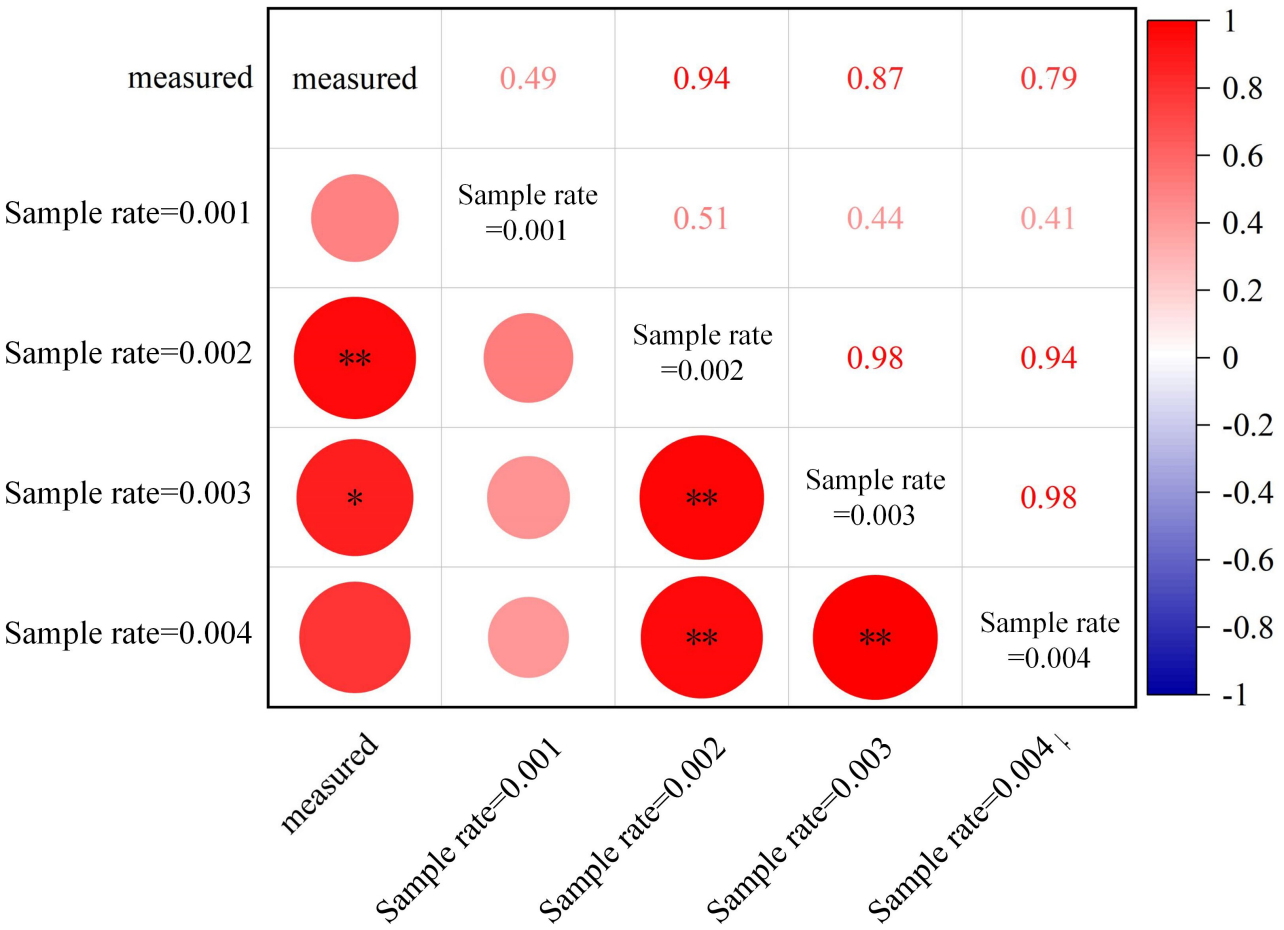
Correlations between automatically extracted root diameters at different sampling rates (cloud parameter, CP) of point clouds from AdQSM and manually measured values. **p < 0.01; *p < 0.05.

Specific algorithms were implemented in AdQSM to quantitatively extract phenotypic parameters (e.g., volume, surface area, diameter, and length) while generating models (**Table 3**). The AdQSM algorithms were optimized by adjusting the height segmentation (HS) value and the cloud parameter (CP). The traits are extracted from the default values; i.e., the HS and the CP are set to 0.5 and 0.003, respectively. The CP represents the degree of point cloud thinning, i.e., down-sampling. **Figure 9** reveals the variation of branch volume, branch surface area, the average RD, and branch length with different CPs (range from 0.001 to 0.004). The figure indicates that the RD generally increases with the increase of CP values, while the root length decreases. The diameter and length of the roots showed opposite trends, resulting in an irregular variation of area and volume obtained by the balanced calculation of these two. **Figure 10** shows the correlation analysis between the extracted RD at different CP values and the manually measured values. The extracted diameter values were significantly correlated with the measured values at CP values of 0.002 (*r* = 0.94, *p* < 0.01) and 0.003 (*r* = 0.87, *p* < 0.05). Therefore, it can be judged that 0.002 is the optimal CP value in this study. The CP values were directly related to the final extraction accuracy of the RD. Careful point cloud thinning is essential for reducing data redundancy and facilitating the accurate extraction of structural parameters. It is important to note that, given that only the diameter has been measured and other extracted characteristics were unavailable to be verified, the optimal value of CP should be considered purely as a reference point. The approach needs the coordinate file of the input point clouds to generate the model and finishes extracting phenotypes automatically. According to **Table 3**, it can be seen that the model’s extracted diameters are smaller than those measured ones.

**Table 3:** The phenotypic parameters of roots automatically extracted by the AdQSM method.

Tree ID	Number of roots ± SE	Measured diameter ± SE (cm)	Parameters for automatic extraction			
			Extracted diameter ± SE (cm)	Root volume (m^3^)	Root surface area (m^2^)	Root length (m)
**a**	30 ± 3	4.37 ± 0.57	3.73 ± 0.62	0.01 ± 0.00	2.06 ± 0.35	108.11 ± 10.21
**b**	52 ± 5	4.07 ± 0.37	2.77 ± 0.86	0.02 ± 0.00	3.36 ± 0.36	281.25 ± 16.58
**c**	28 ± 2	7.75 ± 0.88	5.44 ± 1.02	0.05 ± 0.01	6.59 ± 0.77	248.29 ± 13.57
**d**	32 ± 4	3.60 ± 0.82	2.50 ± 0.97	0.02 ± 0.00	2.44 ± 0.28	175.88 ± 14.23
**e**	55 ± 3	4.66 ± 0.35	4.51 ± 0.69	0.05 ± 0.01	7.39 ± 1.04	365.02 ± 18.69
**f**	19 ± 2	5.21 ± 1.15	3.70 ± 1.54	0.04 ± 0.01	4.57 ± 0.83	234.83 ± 15.47

SE refers to standard error. Each data value denotes the mean ± SE. The number of roots here represents only the number of the taproot and the first- and second-order lateral roots.

## 4 Discussion

The 3D point clouds from TLS show that the spatial distribution of the *Ginkgo* root system extends further horizontally than vertically, which is consistent with the results of Men (1986). Although the *Ginkgo*’s taproot was demonstrated to be distinguishable, certain sample trees lost their taproot dominance, possibly as a result of environmental factors such soil depth or water table, which, in turn, altered the root system’s hierarchical structure (Freschet et al., 2021a). Liu et al. (2007) established a 3D static model of the root system of *Pinus tabuliformis* Carr. based on fractal theory. It concluded that there is a strong correlation between the RD and the root length, which can be used to predict the RD. However, this requires much more time and labor to measure the relevant parameters to establish the relationship model, which is complicated for tree species with more root branches. Guo et al. (2008) revealed a strong correlation between the RD and the root branch order. According to Li et al. (2016), the RD can be used to infer root biomass, while its variation is driven by soil, water, and nutrients. Conventional measurements of root system morphological and structural parameters are typically labor-intensive and time-consuming. Quantitative description of the link between phenotyping traits and the function of roots has been the endeavor of many scholars. van Dusschoten et al. (2016) estimated root length in different RD classes for maize based on MRI images and established linear regression relationships with an *R* ^2^ and RMSE of 0.66 and 0.68 cm, respectively. In this study, the *R* ^2^ and RMSE of the RD of *Ginkgo* derived from TLS were 0.99 and 0.47 cm, respectively, which were highly consistent with the measured values and can substitute manual measurement. MRI is usually performed indoors, and the TLS is relatively flexible in the sites of use and less labor-intensive.

Zhu et al. (2014) employed GPR to obtain 3D images of pine root systems and indirectly estimate the underground biomass by establishing the model of RD. It took 2 to 3 h for GPR to scan a root system and could vaguely distinguish the root distribution position from an image with a low resolution. The results demonstrated that the RD error was between 13% and 16%, when the best RD estimation model was tested against the measured data. The electromagnetic waves emitted by GPR are affected by the dielectric constant. The resolution of the medium is mainly adopted to detect coarse roots and their distribution. Miao et al. (2022) utilized TLS to scan maize, convert point clouds into images, and assess stem thickness using elliptical fitting. The results achieved high accuracy, allowing for the quick determination of crop phenotypic traits. However, when 3D point clouds are turned into 2D images for measurement, it not only increases the source of error and decreases measurement accuracy, but also lengthens the data processing time. Furthermore, because there were more sites to scan, it took longer and increased the risk of error (Disney, 2019). In this study, the root system can be scanned by TLS to directly acquire the millimeter-level accurate 3D morphological structure and realistic texture information of the root system. The five-station scanning method takes approximately 20 min to obtain the object’s high-density point clouds. In addition, the TLS point cloud-based measurements in this study improved at least 30% in terms of efficiency and achieved a higher level of accuracy. The 3D visualization of root systems and model reconstruction is essential for understanding the morphology, structure, and function of plant root systems (Wu et al., 2021a). Despite the fact that there are various ways for modeling roots, we still require a systematic approach as most have limitations and are not very universal. Studies on 3D model visualization of roots mostly focus on studying monocotyledons (Delory et al., 2022). Han and Kuo (2018) constructed a 3D image of the rice root system and quantified phenotypic traits, such as lateral root number and surface area.

To explore the dynamic growth process of the rice root system, Yang et al. (2020) proposed a 3D growth model based on a differential L-system. The model fitted the total root length and surface area to the measured values with an accuracy of more than 95%. In this study, woody plant roots were investigated. The results of this study indicated that the software for single-tree modeling could be implemented to root modeling, through the coordinate transformation to represent the complex architecture of roots completely. Regarding the RD extraction, the RMSE of the diameter was controlled within 2 to 3 cm for both models, and the models were well-performed. The model value of the RD is generally higher than the measured value. The overestimation may be caused by the misalignment of the point clouds, the incorrect recognition during modeling, and the mistaking noise as a component. By improving the registration accuracy and precise denoising, these issues can be alleviated and the model accuracy can be increased. In terms of root detection results, the MST-based and TIN-based models could correctly detect most first-order and second-order lateral roots. The results showed that the overall accuracy of the MST-based model (F1 = 0.81, Accuracy = 0.83) was slightly higher than that of the TIN-based model (F1 = 0.80, Accuracy = 0.82). The F1-score values of second-order lateral roots were higher than those of the first-order ones. Specifically, the sample tree labeled e (total number of roots is 55) had the highest accuracy, while the sample d (total number of roots is 32) had the lowest accuracy. These may be because the larger the number of roots, the wider the spatial distribution area, and the easier to be scanned by the laser, thereby weakening the influence of environmental factors and improving the detection accuracy.

Yang (2021) utilized SketchUp for 3D modeling of slope protection plants, and the three root architecture parameters extracted by the model were highly linearly correlated with the true values. The study required manual measurement of RDs and coordinates, which was relatively time-consuming and laborious. In this study, root scanning using TLS can obtain not only the 3D structure of the root system, but also the true color image as well as the coordinates. This can reduce the error of human measurement. Though the two models in this study performed worse than SketchUp, automatic modeling makes modeling reasonably straightforward (only takes 3–15 min) and enhances the efficiency of digitization processing. The possible reasons for the lower accuracy are that the model algorithm is developed mainly for the aboveground part of the plant and is prone to systematic errors. Alternatively, this was caused by the influence of environmental factors in scanning the root system, which resulted in point cloud noise. Hui et al. (2022) extracted the number of first-order lateral roots by segmenting and scanning the image. However, this method is time-consuming and cannot directly capture the true 3D spatial distribution structure of the root system, as it is based on 2D images. Compared with other methods, the millimeter resolution and high penetration of TLS can accurately capture the structure and texture of the root system. Specific algorithms can also be implemented for the 3D reconstruction of the entire root system to quantify more traits quickly, efficiently, and with high throughput. The 3D reconstruction of root systems based on point clouds overcomes the limitation of traditional 2D image-based modeling with a single perspective.

Nevertheless, this study also has some limitations. The number of samples should be expanded, and differences in age, tree species, site conditions, and culture methods (cuttings and seedlings) of the samples should be taken into account. Currently, this paper is based on a semi-automated method for extracting the parameters of a 3D root model. The approaches primarily focus on separating and reconstructing branches and leaves aboveground. However, it is recommended that future research focuses on developing specialized algorithms for the separation and reconstruction of 3D root systems to enable automatic extraction of root parameters. The lack of measured data on root length in this study made it unavailable to validate the automated extraction of phenotypic traits, such as surface area and volume. In this study, fine roots less than 5 cm in diameter were clipped, due to their potential to introduce noise into the point cloud data. Consequently, this exclusion led to the loss of characterization of the fine roots and obscured their contribution to traits, such as surface area and the total root length. Owing to the presence of noise caused by fine roots in the point cloud, the accuracy analysis presented in this study has been restricted to the first-order lateral roots (including the tap root) and the second-order lateral roots. This study serves as an initial application of tree modeling methods for the extraction of phenotypic parameters of roots. We aim to validate further levels of roots using this approach in the future, thus optimizing the model and enhancing its robustness. This study provides a technical reference for the extraction of 3D root structure parameters of other trees. Refined extraction of root phenotypes can help improve our understanding of carbon and nitrogen allocation in tree organs and potentially improve future forest genetic gains. Based on TLS, it is hoped that future researchers will continue to develop methodological techniques to reconstruct tree root systems and be able to automatically extract phenotyping traits. That would be crucial for evaluating the analysis of spatial distribution structure, forest biomass, and growth structure for trees. This will facilitate improved understanding of precise plant cultivation, integration of phenotypes and genotypes, exploration of physiological and biochemical plant properties, and enhanced mechanical anchor of root systems.

## 5 Conclusion

There is still a lack of high-throughput data collection and modeling approach for root systems of trees. In this study, a new approach for quantifying root phenotyping based on ARPM was developed. This approach provides a potential avenue for improving 3D modeling algorithms and offers a new impetus for root phenotyping measurements. High-precision TLS point clouds can access sophisticated 3D structures of the root system. Compared to existing methods, the developed ARPM approach offers numerous advantages, including wider site applicability, reduced time and labor costs, and increased data collection and analysis efficiency and accuracy. Fitting of the diameter and the number of lateral roots showed that TLS is a reliable means to obtain root information effectively with high accuracies. The reconstructed models based on point clouds can not only present the spatial distribution and topology of the root system but also quantitatively extract the corresponding phenotyping traits.

## Data availability statement

The raw data supporting the conclusions of this article will be made available by the authors, without undue reservation.

## Author contributions

YL: Data curation, Formal analysis, Investigation, Methodology, Software, Validation, Visualization, Writing – original draft, Writing – review & editing. KZ: Conceptualization, Data curation, Formal analysis, Funding acquisition, Investigation, Methodology, Validation, Writing – original draft, Writing – review & editing. LC: Conceptualization, Project administration, Resources, Supervision, Writing – review & editing.

.
